# A *katE katG* double-knockout *E. coli* strain eliminates the risk of catalase contamination in recombinant proteins

**DOI:** 10.1007/s00253-026-13820-2

**Published:** 2026-04-22

**Authors:** Axel Tobias Scholz, Lucia Coppo, Edward Nolan, Michaella Hernandez, Xuan Wang, Pradeep Mishra, Robert Schnell, Zsuzsanna Anna Pató, Yifei Chen, Markus Dagnell, Attila Andor, Qing Cheng, Elias S. J. Arnér

**Affiliations:** 1 Department of Medical Biochemistry and Biophysics, Division of Biochemistry, Karolinska Institutet, SE-171 77, Stockholm, Sweden; 2 School of Life Sciences, Arizona State University, Tempe, AZ USA; 3 Department of Medical Biochemistry and Biophysics, Umeå University, SE, 901 87 Umeå, Sweden; 4 Department of Neuroscience, Karolinska Institutet, SE, 171 77 Stockholm, Sweden; 5 Department of Molecular Neurosciences, Center for Brain Research, Medical University of Vienna, Spitalgasse 4, 1090 Vienna, Austria; 6 Department of Selenoprotein Research, National Tumor Biology Laboratory, National Institute of Oncology, Ráth György str. 7–9, Budapest, HU-1122 Hungary; 7 The Second Affiliated Hospital Zhejiang University School of Medicine, Hangzhou, China; 8 Present Address: NorthX Biologics AB, Sundsvall, Sweden

**Keywords:** Recombinant protein expression, *Escherichia coli* (*E. coli*), Protein purification, Hydrogen peroxide, Catalase, Peroxiredoxin, Thioredoxin

## Abstract

**Abstract:**

Recombinant protein expression in *E. coli* is a key methodology for modern biomedical research. Typically, a polyhistidine-tagged (“His-tagged”) protein is purified using immobilized metal affinity chromatography (IMAC), achieving close to apparently homogenous target protein preparations. However, contaminant host proteins may nonetheless be co-purified at trace amounts. This includes bacterial catalase, which can even be found crystallized instead of an intended target protein. Here, we found that less than 0.03% of the original endogenous bacterial catalase remaining in a final recombinant protein product can easily be detected in an enzymatic H_2_O_2_ (hydrogen peroxide) scavenging assay, because of the high inherent turnover of catalase and its lack of need for additional cofactors. If present in a recombinant protein preparation, this activity may give unintended effects, especially if the target protein is a redox active enzyme, such as glutathione peroxidase, glutaredoxin, ribonucleotide reductase, thioredoxin, or peroxiredoxin. Here, we found that genetic deletion of the two *katG* and *katE* genes in a bacterial expression host could fully eliminate catalase from the recombinant protein product without any appreciable loss of final yield. We suggest that this genetic approach is to be preferred for the removal of catalase instead of using more extensive purification schemes.

**Key points:**

• *Catalase contaminates recombinant His-tagged proteins purified from E. coli.*

• *A small amount of catalase yields substantial activity due to its high turnover.*

• *Genetic knockout eliminates catalase contamination without compromising yields.*

**Graphical Abstract:**

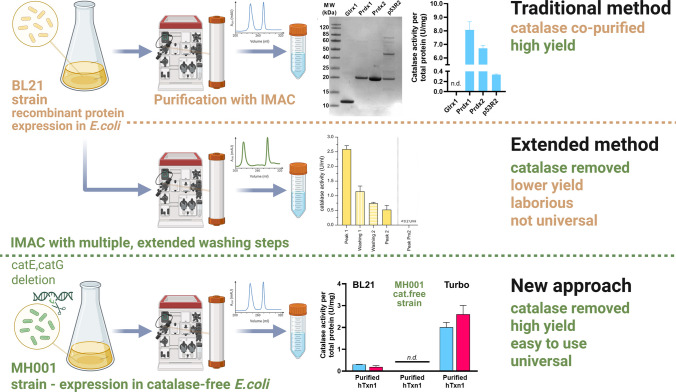

**Supplementary Information:**

The online version contains supplementary material available at 10.1007/s00253-026-13820-2.

## Introduction

Incorporating a string of histidine residues (a “His-tag”) at the C- or N-terminal ends of a protein overexpressed in *Escherichia coli* (*E. coli*) for subsequent purification via an immobilized metal affinity chromatography (IMAC) has become the main method for production of recombinant proteins, which has revolutionized the field of protein biochemistry (Gräslund et al. 2008 ; Schmitt et al. 1993; Hochuli et al. 1988; Porath et al. 1975). Immobilized metal ion matrices, such as nickel–nitrilotriacetic acid (Ni–NTA), are used in this process since they tightly bind the His-tag (Schmitt et al. 1993). However, some native *E. coli* proteins can also bind to the immobilized metal, potentially leading to contamination of the final product (Schmitt et al. 1993; Arnold 1991; Bolanos-Garcia and Davies 2006). While most such contaminants, typically present at trace amount levels, may not interfere with downstream investigations of the target protein, there may be important exceptions. One such potential exception could be co-purified bacterial catalase (Grzechowiak et al. 2021), which we address in this study. Catalase is a tetrameric hemoprotein that converts hydrogen peroxide (H_2_O_2_) into water and oxygen without the need of additional cofactors. The enzyme is expressed in all aerobic organisms and, as noted as early as 1900 by Oscar Loew, “There seems to exist no plant and no animal which is without that peculiar enzyme, which the writer proposes to name catalase from its catalytic action on hydrogen peroxide.” (Loew 1900). *E. coli* has two catalase-encoding genes, *katE* and *katG* (Doukyu and Taguchi 2021). Catalase is, furthermore, one of the most potent enzyme catalysts known; see Smejkal and Kakumanu for a recent review (Smejkal and Kakumanu 2019). H_2_O_2_, a molecule which is involved in both oxidative stress and cellular signaling events, is capable of directly interacting with a number of redox-sensitive proteins, also under physiological conditions (Sies et al. 2022). Hence, if a redox-sensitive target protein is produced in a recombinant form, trace amounts of catalase in such a protein preparation can significantly impact downstream studies, especially if effects of H_2_O_2_ are to be studied.

Several genetically engineered bacterial strains have previously been developed to tackle the most abundant and common impurities found in recombinantly expressed proteins, namely, the bacterial *slyD*, *arnA*, *can*, *aceE*, and *glmS* gene products; here, the approaches have been to change histidine residues in these proteins to serine or alanine, eliminating their histidine-rich domains, or by adding chitin binding domain (CBD)-tags for separate exclusion with a chitin-column step (Robichon et al. 2011; Andersen et al. 2013). Other approaches specifically avoiding co-purification of SlyD have included the engineering of a *slyD*-deficient *E. coli* strain or the targeting of SlyD with nanobodies (Mokhonov et al. 2018; Hu et al. 2017). However, these approaches did not specifically address potential co-purification of bacterial catalase. Here, we characterized the extent of catalase co-purification with different target proteins purified using routine purification schemes. We finally constructed and evaluated a catalase-deficient *E. coli* host strain for the potential production of recombinant proteins completely lacking catalase. The results reveal that bacterial catalase easily becomes co-purified at trace amounts when using popular expression host strains, such as BL21(DE3) or Turbo, whereas using the new catalase-deficient strain eliminates that outcome while nonetheless resulting in comparable yield and purity of the target protein.

## Experimental procedures

### Chemicals and reagents

All chemicals and reagents were of high purity and obtained from regular larger vendors of chemicals and reagents, such as Sigma-Aldrich, Thermo Fisher, or Merck, unless specifically noted otherwise.

### *E. coli* strains and plasmids

The strains and plasmids used in this study are summarized in Table 1, together with references to their origin, or indicated as “This study” when developed herein.

**Table 1:** Specific *Eschericia coli* strains, plasmids, and primers used in this study

	Relevant characteristics	Reference
***Eschericia coli* strains**		
BL21(DE3)	Commercial protein production host strain	Thermo Fisher
Turbo	Commercial cloning strain	NEB
C321.ΔA	RF1-deficient *E. coli* MG1655 strain with all TAG termination codons removed	Lajoie et al*.* (Lajoie et al. 2013), obtained from Addgene
BW25113	Widely used K-12 laboratory strain, used as the parent strain of the Keio collection	(Grenier et al. 2014; Datsenko and Wanner 2000)
MH001	BW25113 *katG*::FRT *katE*::FRT	This study
**Plasmids**		
pKD46	A plasmid for Red recombinase-catalyzed site-targeted modification of genomic loci in *E. coli*	Datsenko & Wanner (Datsenko and Wanner 2000)
pCP20	Plasmid for temperature-sensitive replication and thermal induction of FLP expression	Datsenko & Wanner (Datsenko and Wanner 2000)
pD441a-HTXN1^*^	Expression plasmid for human TXN1	Cheng et al. (Cheng and Arnér 2017)
pD441a-HPRDX2^*^	Expression plasmid for human PRDX2	This study
pD441a-HPRDX1^*^	Expression plasmid for human PRDX1	This study
pD441a-HGLRX1^*^	Expression plasmid for human GLRX1	This study
pD441a-HRRM2^*^	Expression plasmid for human RRM2	Tran et al. (Tran et al. 2024)
pNIC-His6NirBD^*^	Expression plasmid for M.tb. NirBD	Expression vector based on pNIC28Bsa4 (GenBank Acc. Nr. EF198106)
pBAD-CysG	Expression plasmid for S.t. CysG	Schnell et al. (18)
**Primers for genotyping *kat* targeting**		
***katE*** up	ATAATCTGGCGGTTTTGCTG	This study
***katE*** down	TATCGGTTGGGGAGTTATCG	This study
***katG*** up	AGCCGTGAAGGAGTGAAAGA	This study
***katG*** down	ACGGCATGGTATAGCTCAGG	This study
***BLIC-F1***	TACTTCCAATCCATGCCTACGGCTGGGAGTTC	This study
***DLIC-R***	TATCCACCTTTACTGTTAGACCGCTACCCGCGCGAC	This study

^*^These expression plasmids are driven by the T5 promoter, being recognized by the endogenous RNA polymerase of *E. coli*, thus enabling heterologous protein expression in a variety of *E. coli* strains

### Recombinant protein expression and purification

#### X-ray crystallography purification scheme

A DNA fragment encoding the bicistronic *NirBD* operon (Rv0252 and Rv0253) was amplified by PCR using appropriate primers and *Pfu*-Turbo polymerase (Stratagene) with *M. tuberculosis* H37Rv genomic DNA as template, cloned and ligated to the pNIC28Bsa4 expression vector (GenBank accession no. EF198106) using ligation-independent cloning (Oke et al. 2010). The used expression vector provides an N-terminal His6-tag followed by a TEV-protease site allowing tag-removal (H2N-MHHHHHHSSGVDLGTENLYFQSM), with the tag fused to the N-terminus of the target *NirB* sequence. The previously established co-expression of *CysG* (uroporphyrinogen methyltransferase) for supporting the siroheme co-factor availability during expression and purification of siroheme proteins was used by co-expressing *CysG* from a compatible expression vector under arabinose-control (Schnell et al. 2005; Crane et al. 1995). Protein expression was carried out at 21 °C in 2000 ml LB medium supplemented with kanamycin (30 µg/ml) and chloramphenicol (20 µg/ml). The cultures were grown till mid-log phase (OD_600_ 0.5–0.6) and expression of target protein was induced by addition of L-arabinose (0.02%), FeSO_4_ (1 mM), and IPTG (0.1 mM). The culture continued for 24 h whereupon the cells were harvested by centrifugation and pellets kept frozen at −80 °C until use.

The NirBD complex was purified with a Ni–NTA column (Thermo Scientific) and eluted using an imidazole gradient ranging over 50–500 mM. The fractions containing high amounts of the NirB protein band (200-mM and 300-mM imidazole fractions) were collected, diluted to decrease the NaCl concentration to 150 mM, and loaded on a hand-packed Q-sepharose column equilibrated by 25 mM Tris–HCl pH 8.0 with NaCl (150 mM). Elution was carried out with the same buffer with an increasing NaCl gradient, collecting the brown NirB-containing fractions at the 300–400 mM NaCl concentration range. This protein preparation was concentrated in a Centricon concentrator device (Amicon) with a 10-kDa molecular weight cut-off filter to 1.5 ml and subsequently loaded onto an S200 size exclusion chromatography column (GE-Healthcare). The preparation was equilibrated with the appropriate buffer (25 mM Tris–HCl at pH 8.0, 150 mM NaCl, and 1 mM DTT), and the peak corresponding to the NirBD protein was again collected and concentrated using the Centricon concentrator device (Amicon) with a 10-kDa molecular weight cut-off filter. The small subunit (NirD) was lost during this purification procedure. The final protein preparation in its final buffer (25 mM Tris–HCl at pH 8.0, 150 mM NaCl, 1 mM DTT, and 10% glycerol) was concentrated to 4.4 mg/ml, aliquoted to 55 µl volumes, flash frozen in liquid nitrogen, and kept at −80 °C until further use for crystallization.

#### Regular purification scheme

Plasmids for expression of target recombinant proteins as listed in Table 1 (for sequences and cloning details of PRDX1, PRDX2 and GLRX1, see Supplemental Table S2) were used for transformation of expression the respective *E. coli* host strains. Briefly, overnight cultures of transformed bacteria were inoculated into 2000 ml of terrific broth (TB) medium supplemented with 50 µg/mL kanamycin in a 5000-ml bottle, which was then placed on a shaking incubator at 37 °C. After 3 h, the temperature was reduced to 25 °C and target protein expression was induced by adding 0.5 mM IPTG. The bacteria were subsequently harvested by centrifugation and resuspended in IMAC binding buffer (50 mM Tris–HCl, 100 mM NaCl, 10 mM imidazole, and pH 7.5), followed by sonication for lysis. The soluble fraction was obtained through centrifugation and loaded onto a HisPrep FF 16/10 column attached to an ÄKTA explorer FPLC system (Cytiva Life Sciences). The N-terminal hexahistidine-SUMO tag was then cleaved using ULP1 and filtered out by passing through the column once more, as described previously (Butt et al. 2005). The eluted target protein was concentrated, buffer exchanged to buffer containing 50 mM Tris–HCl (pH 7.5), 100 mM NaCl, 2 mM EDTA, and 20% glycerol, and stored at −20 °C until further analysis. Purity of final proteins as determined by SDS-PAGE typically exceeded 95%.

#### Extensive purification scheme for PRDX2

The recombinant PRDX2 was expressed in *E. coli* C321.ΔA with conditions and purification of the PRDX2 target protein initially mostly following the procedures described above. Briefly, a 40-ml aliquot of overnight culture of the transformed bacteria was used to inoculate 2000 ml terrific broth (TB) media supplemented with 50 µg/ml kanamycin in a 5000-ml bottle. Host C321.ΔA cells (Cheng and Arner 2017) were propagated at 33 °C in shaking incubators and around 5–6 h after inoculation, when the optical density of the cultures reached 1.2–1.4 at 600 nm, the temperature was decreased to 24 °C. Target protein expression was initiated by adding 0.5 mM IPTG and culturing was continued overnight. The bacterial cells were subsequently harvested by centrifugation and suspended in binding buffer for nickel-column affinity chromatography (50 mM Tris–HCl, 250 mM NaCl, 25 mM imidazole, and pH 7.5). Lysis was achieved by sonification, and the cell lysate was centrifuged, with the supernatant containing the soluble fraction of proteins loaded to a HisTrap™ HP 5 ml column (Cytiva Life Sciences) installed on an ÄKTA explorer FPLC system (Cytiva Life Sciences). After washing out the contaminating proteins from the column, a part of the 6xHis-SUMO-PRDX2 and the *E. coli* catalases (HPI and HPII) were eluted from the column under isocratic condition using 40% elution buffer (50 mM Tris–HCl, 250 mM NaCl, 500 mM imidazole, and pH 7.5). This fraction, containing mainly the His-tagged PRDX2 and the host’s catalases, was named Peak1. Then applying 100% elution buffer, the rest of the His-tagged PRDX2 was eluted from the column and that fraction was named Peak2. This chromatographic step, where Peak1 and Peak2 were separated was named the 1 st IMAC. Subsequently, using only Peak2, which contained a small amount of catalase, the fusion tag was cleaved from the N-terminal part of the target protein using 6xHis-ULP1 SUMO protease made in-house. The resulting mixture of target PRDX2, 6xHis-ULP1, and 6xHis-SUMO tag was subsequently reloaded to the same HisTrap™ HP 5 ml column in order to facilitate binding of the His-tagged target protein as well as contaminating proteins binding to the column, with the nontagged PRDX2 being retrieved in the flow-through fraction (named the 2nd IMAC). The same 6xHis-ULP1 SUMO protease digestion followed by separation of PRDX2 target protein from the other proteins present in the fraction was performed also for Peak1, as for Peak2, in order to investigate whether pure, catalase-free PRDX2 could also be obtained from Peak1. After measuring the catalase content of each fraction using the Amplex red^TM^ assay (see below), pure PRDX2 could be obtained from Peak2 fraction of 1 st IMAC only. After buffer change and concentration, the recombinant PRDX2 was stored in TE buffer (50 mM Tris, 2 mM EDTA, and pH 7.5) containing 150 mM NaCl and 30% glycerol. The purity of the isolated proteins was over 95% as judged by SDS-PAGE analysis.

### X-ray crystallography

Protein crystals were obtained by the vapor diffusion method in hanging drop format at 20 °C using crystallization buffer (0.1 M Bis–Tris-Propane at pH 7.75, 0.2 M Na-acetate, and 20% PEG3350). X-ray diffraction data sets were collected at the European Synchrotron Radiation Facility (ESRF) beamline ID29, indexed and integrated using MOSFLM (Leslie 2006), and scaled by AIMLESS from the CCP4i suite (Winn et al. 2011). The structures were solved by molecular replacement using PHASER (McCoy et al. 2007) with the ligand-free coordinates of the *E. coli katE* (PDB: 1CF9) (Mate et al. 1999) as search model. The *katE* structure was completed by manual model building in COOT (Emsley et al. 2010) and interspersed with refinement using PHENIX (Adams et al. 2010). The crystallographic models were validated using COOT and MOLPROBITY (Williams et al. 2018). The model’s B-factors were analyzed with BAVERAGE (Winn et al. 2011). The structure figures were prepared using PyMOL (http://www.pymol.org), with the X-ray diffraction data statistics and model parameters presented in Table S1. The coordinates and diffraction data have been deposited in the PDB with accession code 28WU.

### FOX assay

Ferrous ammonium sulfate (1 mM), xylenol orange (400 μM), sorbitol (400 mM), and H_2_SO_4_ (200 mM) were dissolved in distilled water to prepare the FOX assay reagent, following procedures described elsewhere (Nourooz-Zadeh 1999). A stock concentration of H_2_O_2_ was determined using the extinction coefficient 43.6 M^−1^ cm^−1^ at 240 nm, and a serial dilution of H_2_O_2_ in distilled water ranging from 400 to 0 µM was used to make a H_2_O_2_ standard curve, with 10 µl of H_2_O_2_ standard solutions added to 100 µl of FOX assay reagent in a 96-well plate. After 30 min of incubation protected from light, the plate was read in a TECAN Infinite M200 Pro plate reader measuring absorbance at 560 nm. Samples were handled in the same manner as the standard curve.

### Amplex red™ assay

Amplex red™ (Thermo Fisher) assay reagent was prepared according to the manufacturer’s datasheet. A serial dilution from a H_2_O_2_ stock was prepared in the same manner as for the FOX assay described above, using 10 µl of H_2_O_2_ standard solution added to 100 µl of Amplex red™ assay reagent in a 96-well plate. After 30 min of incubation protected from light, the plate was read in a TECAN Infinite M200 Pro plate reader measuring the absorbance at 560 nm. Samples were handled in the same manner as the standard curve.

### Catalase activity assay

A standard curve of catalase was created measuring the activity of bacterial catalase from *M. lysodeikticus* (Sigma-Aldrich). For this, catalase was diluted in 100 mM Tris (pH 7.5) with 0.1% BSA to concentrations ranging from 0 to 2.2 U/ml, with units defined as 1 µmol of H_2_O_2_ consumed per minute. The reaction was started by adding H_2_O_2_ (150 µM final concentration) to the catalase standard at a proportion of 1:10 (*v*:*v*). At each timepoint, the remaining concentration of H_2_O_2_ was measured by adding a sample of the reaction mix to either FOX or Amplex red™ assay reagent (1:11) in the respective assays. Samples of the fractions collected during the purification of recombinant human thioredoxin 1 as made for Table 2 were analyzed in the same way, with the exception that each respective sample was first diluted to yield an activity within the catalase activity standard curve and desalted, as needed, since the presence of EDTA interferes with the FOX assay.

### Thioredoxin activity assay

The activity of recombinant human thioredoxin was measured as previously described (Arnér and Holmgren 2001). Briefly, recombinant human Sec-containing thioredoxin reductase 1 (10 nM), recombinant human thioredoxin 1 (10 µM), and NADPH (500 µM) were mixed and preincubated at room temperature for 5 min. The assay was started by the addition of bovine insulin (0.16 mM), and the initial rate of decrease of absorbance at 340 nm was determined using a Tecan Infinite M200 Pro plate reader, with units (U) defined as 1 µmol NADPH consumed per minute.

### Gene inactivation in *E. coli*

Gene inactivation was performed in the BW25113 strain using λ-red-mediated one-step inactivation, essentially as previously described (Datsenko and Wanner 2000). Briefly, linear DNA fragments flanked by ca. 250-bp sequences with homology to the region of interest were prepared by PCR for primary integration (FLP recognition target (FRT)-kanamycin cassette (*kan*)-FRT; FRT-*kan*-FRT) (Baba et al. 2006). Corresponding cells from the Keio collection were used as PCR templates (Baba et al. 2006). The *kan* cassette was removed by FLP-promoted recombination using plasmid pCP20. All genetic manipulations were verified using colony PCR. Sequential gene deletions were made in BW25113. All strains, plasmids, and primers used for gene inactivation are listed in Table 1. Colony PCR was performed to genotype deletions of *katE* and *katG* in *Escherichia coli* strain MH001 using DreamTaq Green PCR Master Mixes (Thermo Scientific™). Single colonies of MH001, together with wild-type control *E. coli Turbo,* served as templates. Gene-specific primer pairs (Table 1) targeting *katE* and *katG* were employed to amplify diagnostic fragments spanning the engineered loci (Supplemental Figure S1).

## Results

### Accidental X-ray crystallization of contaminant

**Fig. 1:**
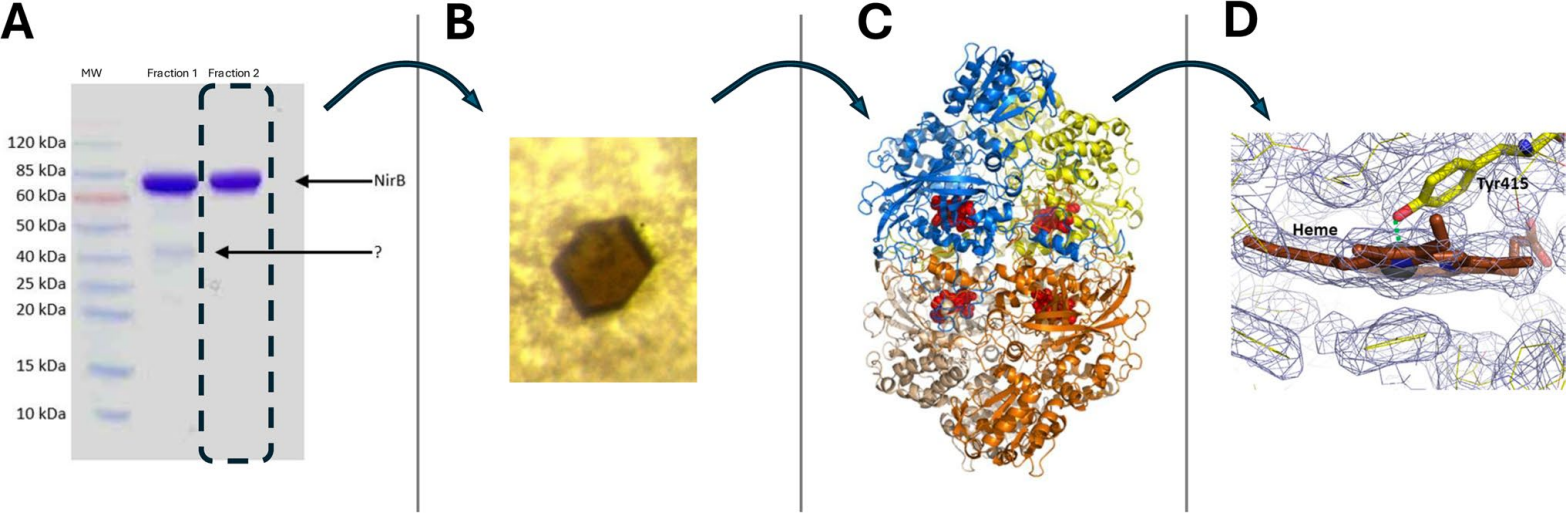
Accidental crystallization of contaminant bacterial host protein *katE*. **A** Coomassie-stained SDS-PAGE of protein preparations from two fractions of the preparative IMAC, containing NirB according to spectral analysis, representing the protein band at ca. 90 kDa as indicated. A contaminating band of approx. 40 kDa was visible in fraction 1, indicated with a question mark, while fraction 2 appeared purified to near homogeneity (dashed box). **B** The protein crystal obtained upon using fraction 2 for crystallization. **C** The homotetrameric structure of *E. coli* HPII (KatE). The subunits are colored blue, yellow, orange, and beige, while the heme cofactor depicted in red. **D** The electron density map contoured at 1σ shown as a grey mesh in the area of the heme-cofactor (brown sticks), with Tyr415 in direct axial contact with the heme iron highlighted (yellow sticks). The coordinates and the diffraction data have been deposited in the PDB with accession code 28WU

**Fig. 2:**
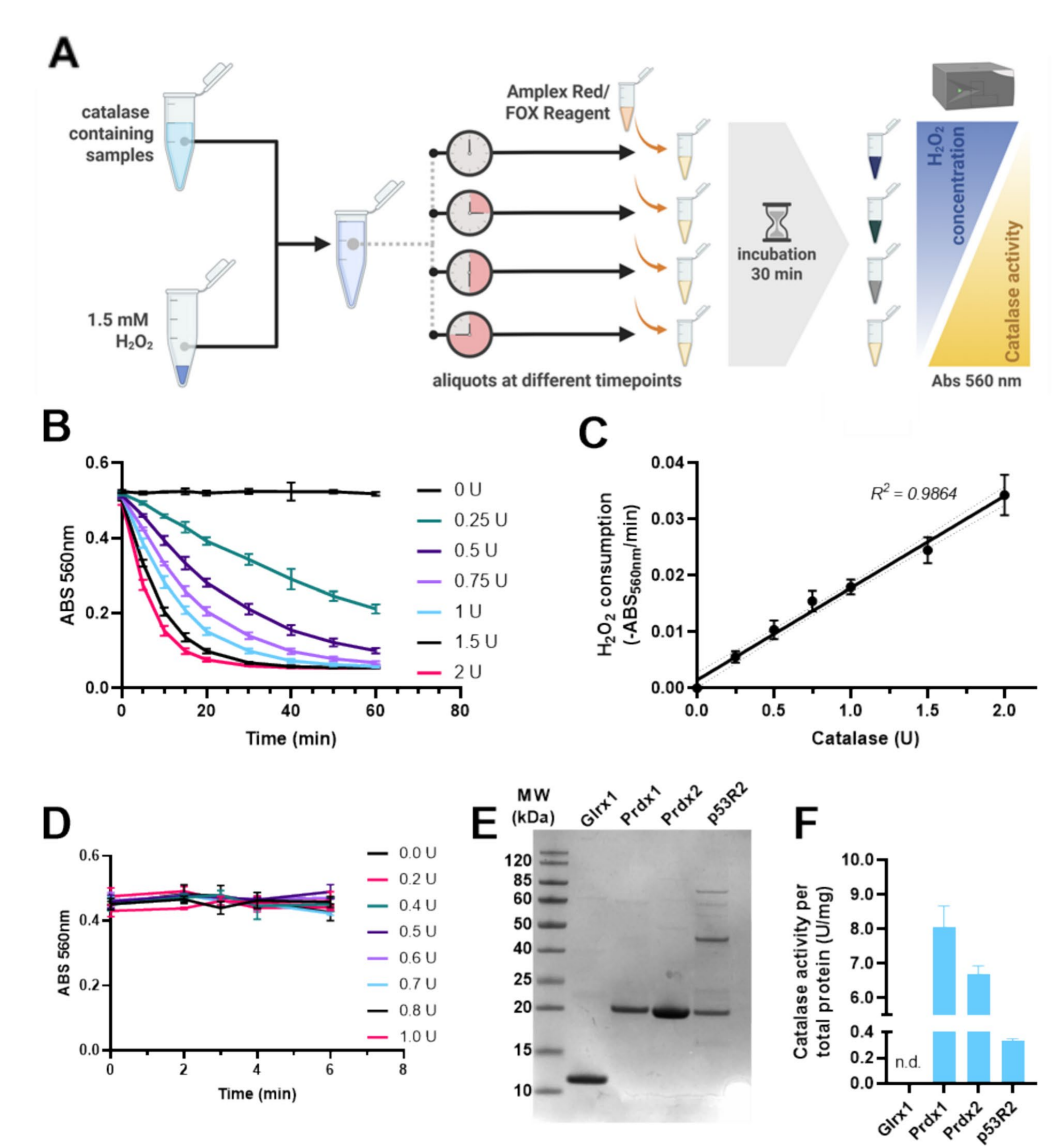
Assessment of catalase presence in recombinant protein preparations. **A** Schematic cartoon of assay for catalase activity determinations. The same pipeline was used with both the FOX and Amplex Red assays, yielding similar results. The FOX assay results are shown here, while the Amplex Red data are in the Supplement. **B** Enzyme kinetics with a catalase standard curve. **C** Standard curve obtained with the FOX assay used to calculate amounts of catalase in recombinant protein samples. **D** Inhibition of catalase activity using NaN_3_ (to be compared with panel **B**. **E** Coomassie-stained SDS-PAGE gel with 10 µg of the indicated recombinant proteins loaded per lane, with any catalase-corresponding band at ca. 40 kDa not being visible. **F **Determination of contaminant catalase activity (U/mg of total protein) in the same protein preparations as those analyzed in (E). n.d. = not detected (< 0.01 U/mg)

This study was initiated upon our accidental identification of bacterial catalase being the crystalized enzyme, when attempting to study the mycobacterial NADPH- and siroheme-dependent nitrite reductase NirBD (Malm et al. 2009). We expressed the enzyme complex in *E. coli* and purified it as outlined in the “Experimental procedures” section, with NirBD consisting of the large subunit NirB (Uniprot: O53674; 90.3 kDa) and the small subunit NirD (Uniprot: O53675; 12.4 kDa). The NirB subunit was successfully expressed and isolated with good purity, as suggested by a gel analysis (Fig. 1A). A spectroscopic analysis furthermore confirmed the presence of the expected cofactors such as siroheme, Fe4-S4 cluster, and FAD, and addition of nitrite ions (NO_2_^−^) produced an absorbance shift of the siroheme-specific peak, according to expectations (data not shown). The protein preparation was hence concentrated, with crystallization screens set up following standard protocols, producing brown crystals that were consistent with our expectations (Fig. 1B), being similar in color to the siroheme proteins NirA and sulfite-reductase that were previously studied (Schnell et al. 2005; Crane et al. 1995). Although the number of crystals and their reproducibility were low, a 2.5 Å diffraction dataset was collected and analyzed. This revealed the P2_1_ space group with unit cell dimensions as *a* = 93.66 Å, *b* = 136.80 Å, *c *= 122.29 Å, and *β* = 112.42° (Supplemental Table S1). Multiple Fe-SAD datasets were evaluated, and extensive molecular replacement trials were attempted, but those were unsuccessful. We thus suspected that the crystals could have originated from a contaminant protein expressed by the *E. coli* host. Molecular replacement trials with Fe-containing *E. coli* proteins were therefore conducted and we found that the unit cell dimensions of the P2_1_ crystal closely matched those of *E. coli* catalase HPII (the *katE* gene product, also named KatE), being *a* = 93.47 Å, *b* = 133.04 Å, *c* = 122.22 Å, and *β* = 109.64°. Indeed, subsequent molecular replacement using the *E. coli* HPII structure (PDB: 1CF9) led to a solution. The electron density map was clear, revealing heme cofactors and showing Tyr415 coordinating the heme’s central Fe-ion by replacing the axial ligand, a characteristic feature among known HPII structures (Fig. 2C and D) (Mate et al. 1999; Bravo et al. 1999). The excellent refinement statistics also confirmed strong agreement with the *E. coli* HPII sequence (Supplemental Table S1), leaving no doubt that the crystals originated from the co-purified *E. coli katE*-encoded catalase.

### Contamination of various recombinant proteins with co-purified host strain catalase

Intrigued by our unexpected finding of crystallizing *E. coli* catalase HPII when present as a contaminant at very low levels, also recognizing that others have reported similar results (Grzechowiak et al. 2021), we next analyzed if additional recombinant proteins produced in our laboratory would have catalase-like enzymatic activity. Expression and purification of the different proteins followed standard expression and purification methods (see “Experimental procedures” section for details). It should be noted here that the presence of catalase in a protein preparation would typically not be detected unless specifically looked for. Thus, we assessed the presence of catalase activity in the protein preparations employing two separate assays for detection of the time-dependent removal of H_2_O_2_, using either ferrous oxidation of xylenol orange (FOX) or oxidation of Amplex red™, as outlined in Fig. 2A and further described in the “Experimental procedures” section (see Supplemental Fig. S1A for linearity of detection with regards to H_2_O_2_ concentrations). We first confirmed that catalase activity could be detected using these assays also when present at very low concentrations, as possibly found in purified proteins, confirming linearity within the ranges used for the analyzed samples (Fig. 2B and C, Supplemental Fig. S1B). For further validation of the assay, we used a known catalase inhibitor, sodium azide (NaN_3_), at a concentration that should fully inhibit the enzyme (Lichstein and Soule 1944), which confirmed that no detectable activity could be measured at any of the tested concentrations of catalase when assayed in presence of NaN_3_ (Fig. 2D). The catalase activity assay could also be inhibited using the alternative inhibitor 3-amino-1.2.4-triazole (3-AT), but NaN_3_ was chosen for this study because of the lower concentrations needed to fully inhibit the enzyme (Supplemental Fig. S1C, S2C-D).

We next used this validated catalase assay to assess the possible presence of catalase in some human recombinant protein preparations purified from *E. coli*, namely, p53-induced subunit of ribonucleotide reductase (p53R2; also named RRM2B), glutaredoxin 1 (GLRX1), peroxiredoxin 1 (PRDX1), and peroxiredoxin 2 (PRDX2), all except p53R2 purified to nearly homogenous purity as judged with a Coomassie-stained SDS-PAGE (Fig. 2E). The analysis revealed that all these protein preparations except GLRX1 contained catalase, with the PRDX1 preparation showing the highest activity of ca. 8 U/mg catalase (Fig. 2F for the FOX assay results; Supplemental Fig. S2D for the Amplex red™ assay).

### Removal of host strain catalase from recombinant PRDX2 using additional purification steps

**Fig. 3:**
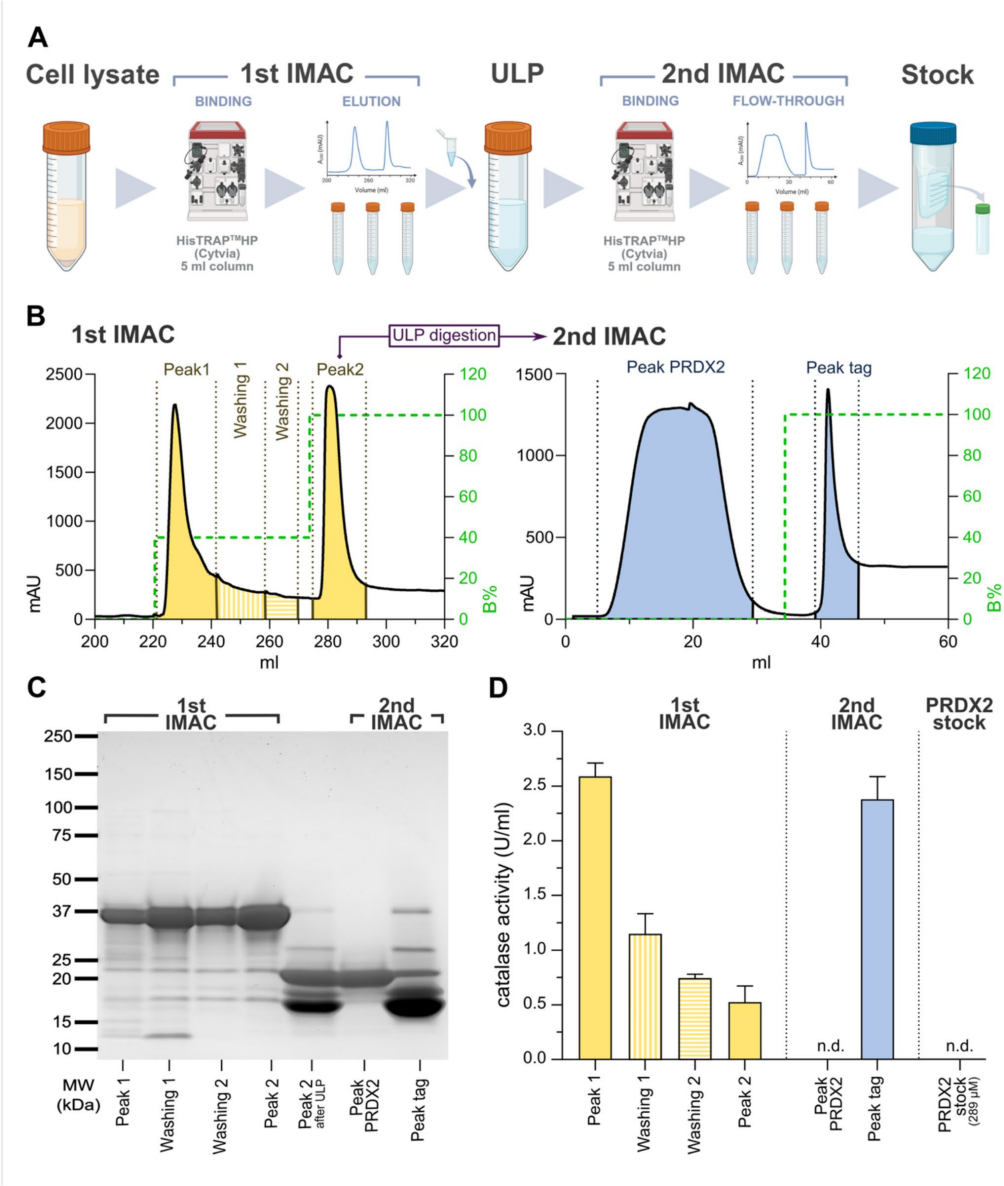
Removal of catalase from recombinant human PRDX2 using a more extensive purification scheme. **A** Graphical illustration of the purification scheme. **B** Chromatogram in yellow of a step-wise elution from the 1st IMAC, resulting in Peak1 and Peak2 preparations as well as washing fractions, as indicated, with Peak2 used for ULP digestion and a second IMAC (blue). B% on the second y-axes refer to the percentage of buffer B (50 mM Tris-HCl, 250 mM NaCl, 500 mM imidazole, pH 7.5) in the eluent, indicated by the dashed green lines. **C** Coomassie-stained SDS-PAGE analysis of the indicated fractions from **A**. **D** Catalase activity in the indicated fractions, as well as in the final concentrated PRDX2 stock solution.

With the different preparations of human recombinant proteins having varying levels of contaminating catalase activity (Fig. 2F), we next attempted to remove the catalase using a more extensive purification scheme, using human PRDX2 as test protein. This is an enzyme where it may be particularly sensitive for subsequent analyses to have contamination of small amounts of catalase, because PRDX2, similarly to catalase, uses H_2_O_2_ as its main substrate (Winterbourn and Hampton 2008). We thus attempted to obtain recombinant PRDX2 using a more elaborate purification procedure over an IMAC column, with more careful elution resulting in two separate peaks of the target protein, as outlined at detail in the "Experimental procedures" and summarized in Fig. 3A. Obtaining Peak1 and Peak2 after the initial purification step (1st IMAC), we subsequently used only Peak2 for ULP digestion of the fusion protein and removal of the digested His-tagged fusion part over a 2nd IMAC (Fig. 3A and B). Analyzing the different fractions for possible catalase contamination, we found that most were of ca. 75–85% purity as judged by SDS-PAGE, except for the final PRDX2 peak, eluting between 5 and 30 ml in the 2nd IMAC (Fig. 3B, left chromatograph), which appeared to have the highest purity (Fig. 3B and C; “Peak PRDX2”). Assessing catalase activity, we found it in all the fractions collected from the 1 st IMAC, as well as in the fraction containing the digested His-tag fusion partner after the 2nd IMAC, while the highly purified PRDX2 after the 2nd IMAC lacked detectable catalase activity (Fig. 3D). Also, upon concentrating that fraction into a final stock of concentrated (289 µM) purified human PRDX2, no catalase activity could be detected (Fig. 3D; “PRDX2 stock”). While this more extensive purification scheme for human PRDX2 hence removed all catalase contamination, the procedure is laborious and furthermore results in a major loss of target protein, as that which ends up in “Peak1” (Fig. 3B) must either be discarded, or again subjected to consecutive IMAC purifications. We therefore next attempted to find another solution for the avoidance of catalase contamination.

### Catalase contamination with human thioredoxin as target protein purified from different *E. coli* hoststrains

To further characterize the possible presence of catalase as contaminant in recombinant protein preparations, we next compared side-by-side the two commonly used BL21(DE3) and Turbo host strains of *E. coli* for overexpression and purification of the same target protein. For this, we used human thioredoxin 1 (hTXN1), representing one of the most common protein folds (Martin 1995), which is furthermore often used as a fusion partner with other recombinant proteins due to its high solubility and ease of expression (Hammarström et al. 2002). We found that also with hTXN1 being the target protein, using the typical IMAC-based procedure for purification from either BL21(DE3) or Turbo as host strains, detectable catalase activity was found in the final protein preparations. We therefore decided to evaluate whether a catalase-deficient *E. coli* host strain could be constructed, which we subsequently compared with BL21(DE3) and Turbo in terms of total yields of hTXN1, as well as evaluating the extent of co-purified catalase activity throughout the purification steps.

**Fig. 4:**
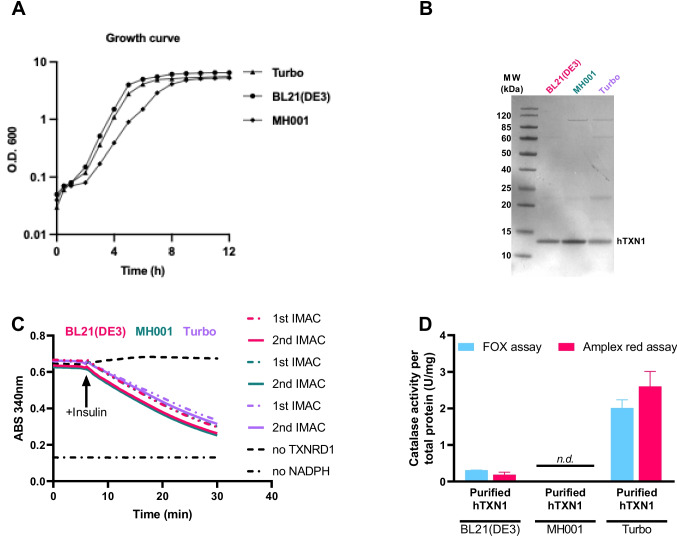
Purification of recombinant human thioredoxin 1 from three different bacteria host strains. **A** Growth curve of the three host strains used. **B** Final protein preparations on SDS-PAGE from the three host strains. **C** Thioredoxin activity in the protein preparations, as assessed using the insulin disulfide reduction assay. **D** Bacterial host catalase activity in the final hTXN1 preparations purified from three different bacterial host strains measured with both FOX and Amplex red™ assays. n.d. = not detected (< 0.01 U/mg)

As previously reported, single knockout strains of *katE* and *katG* have been generated (Datsenko and Wanner 2000), but the double knockout strain was not available to us. We therefore set out to make that strain and were able to construct a fully catalase-deficient *E. coli* by genetic deletion of both the bacterial *katE* and *katG* genes in a BW25113 host strain background using FLP-promoted recombination, validated using colony PCR (Supplemental Fig. S3), here named MH001. Others have shown that catalase deficient *E. coli* are more susceptible to oxidative stress triggered by organic solvents (Doukyu and Taguchi 2021), and we found that MH001 grew slightly slower than Turbo and BL21(DE3) (Fig. 4A), but otherwise it proved easy to handle using normal culturing conditions. MH001 could also be efficiently transformed with the expression plasmid for hTXN1 and used for expression of the recombinant protein. Indeed, when expressing and purifying hTXN1 from Turbo, BL21(DE3) as well as MH001, using the very same procedure with all three strains, we achieved comparable purity of hTXN1 from the three strains (Fig. 4B), also showing similar TXN activities as assessed using a classical mammalian TXN-specific insulin disulfide reduction assay (Arnér et al. 1999) (Fig. 4C). These three preparations were subsequently assessed for catalase activity, which was detected at appreciable levels in the preparations derived from either Turbo or BL21(DE2) but it was, as expected, undetectable in hTXN1 purified from MH001 (Fig. 4D). The protein derived from BL21(DE3) had approximately 0.3 U/mg catalase and that from Turbo had 2 U/mg catalase. Starting from ca. 250-ml crude cell lysates in each case, the total yields of hTXN1 also differed between the three host strains, with ca. 20 mg hTXN1 (1.06 × 10^5^ U) obtained from the Turbo-derived purification, 44 mg (1.57 × 10^5^ U) from MH001 and 53 mg (2.35 × 10^5^ U) from BL21(DE3), representing 21% total yields of the overexpressed hTXN1 from MH001 and Turbo, respectively, and 16% yield in the purification from BL21(DE3). The corresponding yields of bacterial catalase in the final preparations obtained from BL21(DE3) and Turbo were, in contrast, only 0.02% and 0.03%, respectively. Still this was sufficient to yield a total amount of catalase representing 17.1 U and 44.4 U in these two final hTXN1 preparations, respectively. For details in expression, yield, purity, and activities of thioredoxin as well as catalase in all fractions from the three purification schemes, see Table 2.

**Table 2:** Purification table for hTXN1 purification from BL21(DE3), Turbo, and MH001. This table specifies volumes, protein contents, and activities of human TXN1 (hTXN1) as well as bacterial catalase in the major fractions from the corresponding hTXN1 purification steps, as described in the text and summarized in Fig. 4. The hTXN1 activity was measured using the NADPH- and mammalian thioredoxin reductase 1-coupled insulin disulfide reduction assay while catalase activity was determined with the FOX assay and Amplex red™ assays, as outlined in the section

Fractions					Human TXN1					Bacterial catalase			
*E. coli* strain	Fraction	Volume (ml)	Protein (mg/ml)	Total protein (mg)	Estimated total hTXN1 amount (mg)	Total activity (U)	Specific activity (U/mg)	Purification (fold)	Yield (%)	Total activity (U)	Specific activity (U/mg)	Purification (fold)	Yield (%)
Turbo	Crude lysate	255	11.83	3.02 × 10^3^	96.4	5.38 × 10^5^	178	(1)	(100)	1.36 × 10^5^	45.2	(1)	(100)
	Soluble fraction	248	9.67	2.40 × 10^3^	99.4	1.38 × 10^6^	577	3.2	103	1.09 × 10^5^	45.4	1.00	79.83
	Flow-through	260	8.59	2.23 × 10^3^	62.3	6.67 × 10^5^	299	1.7	65	1.07 × 10^5^	48.0	1.06	78.51
	1 st IMAC	25	0.98	24.5	16.3	4.98 × 10^4^	2.03 × 10^3^	11.4	17	39.3	1.60	0.04	0.03
	2nd IMAC	45	0.49	22.1	20.1	1.06 × 10^5^	4.82 × 10^3^	27.0	21	44.4	2.01	0.04	0.03
BL21(DE3)	Crude lysate	257	15.19	3.90 × 10^3^	326	1.01 × 10^6^	259	(1)	(100)	8.15 × 10^4^	20.9	(1)	(100)
	Soluble fraction	250	11.46	2.87 × 10^3^	267	1.50 × 10^6^	522	2.0	82	6.37 × 10^4^	22.2	1.06	78.08
	Flow-through	285	9.54	2.72 × 10^3^	109	8.37 × 10^5^	308	1.2	33	7.96 × 10^4^	29.3	1.40	97.59
	1 st IMAC	35	3.01	105	95.5	3.35 × 10^5^	3.18 × 10^3^	12.3	29	23.2	0.220	0.01	0.03
	2nd IMAC	54	1.03	55.6	53.2	2.35 × 10^5^	4.22 × 10^3^	16.3	16	17.1	0.307	0.01	0.02
MH001	Crude lysate	250	14.80	3.70 × 10^3^	205	1.19 × 10^6^	322	(1)	(100)	*n. d*	*n. d*	***n. d***	*n. d*
	Soluble fraction	250	11.41	2.85 × 10^3^	163	8.30 × 10^5^	291	0.9	80	*n. d*	*n. d*	*n. d*	*n. d*
	Flow-through	281	9.27	2.60 × 10^3^	87.1	5.19 × 10^5^	199	0.6	43	*n. d*	*n. d*	*n. d*	*n. d*
	1 st IMAC	23	3.16	72.7	50.0	1.44 × 10^5^	1.99 × 10^3^	6.2	24	*n. d*	*n. d*	*n. d*	*n. d*
	2nd IMAC	40	1.08	43.2	43.9	1.57 × 10^5^	3.64 × 10^3^	11.3	21	*n. d*	*n. d*	*n. d*	*n. d*

(*n. d.* = not detected)

## Discussion

Expressing His-tagged proteins in bacteria with purification over IMAC is a widely used, cost-efficient method for obtaining recombinant proteins, both for academic research and at an industrial scale. However, potential contamination from bacterial host proteins in the final product is a well-known drawback, recognized decades ago (Hengen 1995). Systematic investigations into the nature of these contaminants have been conducted (Bolanos-Garcia and Davies 2006; Bartlow et al. 2011). Bacterial host catalase as one of the contaminants has also previously been found to be crystalized, in spite of its low amounts in the target protein, similar to our finding herein (Grzechowiak et al. 2021). However, we are not aware of any prior study specifically determining the enzymatic activity of bacterial catalase in recombinant protein preparations, or assessing how best to remove it. We hypothesize that one reason for a lack of directed studies of catalase in recombinant protein preparations may be its typically very low abundance, making it hard to detect unless specifically investigated. Another reason may be the fact that enzymatic removal of H_2_O_2_ within purified protein preparations may sometimes even be beneficial, as a presence of catalase could in fact protect target proteins from oxidative damage during storage.

Here, we first became aware of the phenomenon of catalase co-purification because of the bacterial HPII (the *katE* gene product) having been crystalized, instead of our intended target protein. Our subsequent studies revealed that catalase contamination is typically very low, but not insignificant, and seemed variable despite similar purification schemes as well as initial yields of the intended target proteins. If we consider the catalase contamination in our final hTXN1 preparations as purified from BL21(DE3) and Turbo, the hTXN1 yield, in terms of total units, was 2387-fold higher than that of catalase in the case of Turbo, and 13,740-fold higher than catalase in case of BL21(DE3). However, the presence of *any* catalase in a target protein preparation may still distort possible downstream analyses, especially if such analyses would employ H_2_O_2_ addition, because of the very high catalytic efficacy of catalase that would rapidly assist in removing H_2_O_2_. This could, for example, distort analyses of the activities of recombinantly produced peroxidases, such as peroxiredoxins or glutathione peroxidases. It should however be noted that the choice of assay readout as well as time scale (milliseconds/seconds vs. minutes/hours) will likely affect how much a contaminating catalase would affect the results. For example, determinations of initial rate constants within the millisecond time scale of peroxiredoxins reacting with H_2_O_2_ as measured using alterations in their inherent fluorescence spectra (Kriznik et al. 2020; Nelson et al. 2018) should presumably not be affected by the presence of catalase.

Recent methodological advances, particularly in mass spectrometry, have improved the detection of co-purified bacterial host proteins. Evaluating the results from mass spectrometry-based studies, it is clear that co-purified bacterial catalase is not a problem unique to our research group, as catalase is typically found as a contaminant also in proteins purified and analyzed by other groups (Abeyrathne and Grigorieff 2017; Yantsevich et al. 2017). However, to the best of our knowledge, the enzymatic activity of catalase when present as a contaminant has not been characterized earlier. As already noted, the activity of co-purified contaminating enzymes in recombinant protein preparations can significantly interfere with experimental results and, in some cases, lead to incorrect conclusions. For instance, co-purified bacterial PlsC complicated the study of a mutated human PlsC-like protein by falsely suggesting a failure to modify the recombinant protein—until the issue was resolved using an *E. coli* strain with a *plsC* knockout (McMahon et al. 2014). Another striking example is a prior study, which was later retracted, reporting a novel “catalase-like” activity of PRDX1 (Sun et al. 2015). Although we could not find any explanation for that retraction, it is perhaps likely that co-purified bacterial catalase may have caused misleading results. Similarly, it was proposed that p53R2 has a reactive oxygen species (ROS) scavenger function (Xue et al. 2006), which could possibly be alternatively explained by a contamination of the studied recombinant protein with bacterial catalase. A straightforward method to block a contaminating catalase activity would be to add sodium azide as a potent inhibitor of the enzyme, as we also showed herein. However, sodium azide is toxic (Tat et al. 2021) and has chemical reactivities potentially affecting many different enzyme systems. It is also explosive under certain conditions (Treitler and Leung 2022), meaning that unnecessary use of azide should not be recommended.

In conclusion, here, we have highlighted the importance of considering bacterial host catalase contamination in recombinant protein preparations. Such co-purified catalase may yield unwanted side reactivities even if present at only very small trace amounts, especially if the target protein in question is to be used in redox-sensitive experiments employing H_2_O_2_. Importantly, we here constructed a new catalase-deficient *E. coli* strain and found that it can still support efficient recombinant protein expression, thus effectively mitigating the risk of catalase contamination in target protein products.

## Supplementary Information

Below is the link to the electronic supplementary material.

ESM1 (505 KB)

## Data Availability

X-ray crystallography data is available from the PDB under accession code 28WU.
