# Supplementary material for: A katE katG double-knockout E. coli strain eliminates the risk of catalase contamination in recombinant proteins
Source: Appl Microbiol Biotechnol. 2026 Apr 22;110(1):136. doi: 10.1007/s00253-026-13820-2 (PMC13102901; doi:10.1007/s00253-026-13820-2)
Supplement: Supplementary file 1 — (505 KB) [file 253_2026_13820_MOESM1_ESM.pdf]

Supplementary Information

A *katE katG* double-knockout *E. coli* strain eliminates the risk of catalase contamination in recombinant proteins

Axel Tobias Scholz[1], Lucia Coppo[1], Edward Nolan[2], Michaella Hernandez[2], Xuan Wang[2], Pradeep Mishra[3], Robert Schnell[4,5], Zsuzsanna Anna Pató[1,6], Yifei Chen[1,7], Markus Dagnell[1], Attila Andor[1,6], Qing Cheng[1], Elias S.J. Arnér[1,6*]

[1] Division of Biochemistry. Department of Medical Biochemistry and Biophysics. Karolinska Institutet. SE-171 77. Stockholm. Sweden.

[2] School of Life Sciences. Arizona State University. Tempe. Arizona. USA

[3] Department of Medical Biochemistry and Biophysics. Umeå University. SE-901 87 Umeå. Sweden.

[4] Department of Neuroscience. Karolinska Institutet. SE-171 77 Stockholm. Sweden.

[5] Department of Molecular Neurosciences, Center for Brain Research, Medical University of Vienna, Spitalgasse 4, A-1090 Vienna, Austria

[6] Department of Selenoprotein Research and the National Tumor Biology Laboratory. National Institute of Oncology. Budapest. Hungary.

[7] Current affiliation: The Second Affiliated Hospital Zhejiang University School of Medicine, Hangzhou, China.

* Corresponding author. elias.arner@ki.se

Supplemental Table S1. X-ray diffraction data and refinement statistics for the HPII (KatE) crystal.

Dataset	**E.coli KatE (crystallization by accident)**
Beamline	ESRF / ID29-1
Space group	$P2_1$
Unit cell	
a,b,c (Å)	93.66 136.80 122.30
α,β,γ(°)	90.00 112.42 90.00
Resolution (Å)	41.28-2.50 (2.54-2.50)
No. of unique reflections	92488 (4690)
$I/\sigma(I)$	7.0 (2.2)
Redundancy	2.4 (2.4)
Completeness (%)	94.6 (96.5)
R_{merge}	0.143 (0.654)
R_{pim}	0.103 (0.454)
CC(1/2)	0.978 (0.614)
Wilson B- value (Å2)	32.3
Values in parenthesis are for the highest resolution shell.	
Refinement	
R	0.1597
R_{free}	0.2236
Number of atoms / B-factor A^2	
Overall	24172 / 23.529
Protein	22944 / 23.592
Ligand	172 / 14.375 (heme)
Water	1056 / 23.638
Rmsd from ideal geometry	
Bond length (Å)	0.009
Bond angles (deg.)	1.001
Ramachandran Plot (%)	
Residues in preferred regions	2753 (95.09%)
Residues in allowed regions	125 (4.32%)
Outliers	17 (0.59%)

Supplemental Table S2. Cloning of human PRDX1, PRDX2 and GLRX1 expression construct used in this study. Recombinant human PRDX1, PRDX2 and GLRx1 were here cloned after codon optimization for *E. coli* expression, using gene cassette inserts ordered from Integrated DNA Technologies, Inc. (IDT), with the synthetic genes transferred into the pD441 plasmid developed previously by us (33), to generate fusion proteins linked with an N-terminal His-tagged Sumo protein (for plasmids used, see Table 2). The open reading frames (ORFs) given in the table are flanked by their start (ATG) and stop (TAA) codons, with the resulting protein sequences given in the second column of the table. The final expression plasmids were transformed into various *E.coli* strains for protein production, as described in the text.

**Protein**	**Synthetic DNA Sequence**	**Protein Sequence**
**Human PRDX1**	ATGAGCAGCGGTAATGCAAAAATTGGTCATCCGGCACCGAACTTT<br>AAAGCAACCGCAGTTATGCCGGATGGTCAGTTTAAAGATATTTCC<br>CTGAGCGACTACAAAGGCAAATATGTGGTGTTTTTTTTCTACCCGC<br>TGGATTTTACCTTTGTTTGTCCGACCGAAATTATCGCATTTAGCGAT<br>CGTGCAGAAGAGTTCAAAAAACTGAATTGCCAGGTTATTGGTGCA<br>AGCGTTGATAGCCATTTTTGTCATCTGGCATGGGTTAATACCCCGA<br>AAAAACAAGGTGGTCTGGGTCCGATGAATATTCCGCTGGTTAGCG<br>ATCCGAAACGTACCATTGCACAGGATTATGGTGTTCTGAAAGCAG<br>ATGAAGGTATTAGCTTTCGTGGCCTGTTTATTATCGATGACAAAGGT<br>ATTCTGCGTCAGATTACCGTTAATGATCTGCCGGTTGGTCGTAGC<br>GTTGATGAAACCCTGCGTCTGGTTCAGGCATTTCAGTTTACCGATA<br>AACATGGTGAAGTTTGTCCGGCAGGTTGGAAACCGGGTAGCGAT<br>ACCATTAAACCGGATGTTCAGAAAAGCAAAGAGTACTTCAGCAAG<br>CAGAAATAA	MSSGNAKIGHPAPNF<br>KATAVMPDGQFKDIS<br>LSDYKGKYVVFFFYPL<br>DFTFVCPTEIIAFSDRA<br>EEFKKLNCQVIGASV<br>DSHFCHLAWVNTPK<br>KQGGLGPMNIPLVSD<br>PKRTIAQDYGVLKADE<br>GISFRGLFIIDDKGILR<br>QITVNDLPVGRSVDE<br>TLRLVQAFQFTDKHG<br>EVCPAGWKPGSDTIK<br>PDVQKSKEYFSKQK-

**Human**<br>**PRDX2**	ATGGCCTCCGGTAACGCGCGCATCGGCAAACCGGCCCCGGAC<br>TTCAAAGCCACCGCGGTGGTTGATGGCGCCTTCAAAGAGGTGAA<br>GCTGTCGGACTACAAAGGGAAGTACGTGGTCCTCTTTTTCTACCC<br>TCTGGACTTCACTTTTGTGTGCCCCACCGAGATCATCGCGTTCAG<br>CAACCGTGCAGAGGACTTCCGCAAGCTGGGCTGTGAAGTGCTG<br>GGCGTCTCGGTGGACTCTCAGTTCACCCACCTGGCTTGGATCAA<br>CACCCCCCGGAAAGAGGGAGGCTTGGGCCCCCTGAACATCCC<br>CCTGCTTGCTGACGTGACCAGACGCTTGTCTGAGGATTACGGCG<br>TGCTGAAAACAGATGAGGGCATTGCCTACAGGGGCCTCTTTATCA<br>TCGATGGCAAGGGTGTCCTTCGCCAGATCACTGTTAATGATTTGC<br>CTGTGGGACGCTCCGTGGATGAGGCTCTGCGGCTGGTCCAGGC<br>CTTCCAGTACACAGACGAGCATGGGGAAGTTTGTCCCGCTGGCT<br>GGAAGCCTGGCAGTGACACGATTAAGCCCAACGTGGATGACAG<br>CAAGGAATATTTCTCCAAACACAATTAA	MASGNARIGKPAPDF<br>KATAVVDGAFKEVKLS<br>DYKGKYVVLFFYPLDF<br>TFVCPTEIIAFSNRAED<br>FRKLGCEVLGVSVDS<br>QFTHLAWINTPRKEG<br>GLGPLNIPLLADVTRR<br>LSEDYGVLKTDEGIAY<br>RGLFIIDGKGVLRQITV<br>NDLPVGRSVDEALRL<br>VQAFQYTDEHGEVCP<br>AGWKPGSDTIKPNVD<br>DSKEYFSKHN-
**Human**<br>**GLRX1**	ATGGCCCAAGAATTTGTGAACTGCAAAATTCAGCCTGGTAAAGTG<br>GTGGTTTTTATCAAACCGACCTGTCCGTATTGTCGTCGTGCACAAG<br>AAATTCTGAGCCAGCTGCCGATTAAACAGGGTCTGCTGGAATTTG<br>TTGATATTACCGCAACCAATCACACCAACGAAATTCAGGATTATCT<br>GCAGCAGCTGACCGGTGCACGTACCGTTCCGCGTGTTTTTATTG<br>GTAAAGATTGTATTGGTGGTTGCAGCGATCTGGTTAGCCTGCAGC<br>AGAGCGGTGAACTGCTGACCCGTCTGAAACAAATTGGTGCACTG<br>CAGTAA	MAQEFVNCKIQPGKV<br>VVFIKPTCPYCRRAQE<br>ILSQLPIKQGLLEFVDI<br>TATNHTNEIQDYLQQ<br>LTGARTVPRVFIGKDC<br>IGGCSDLVSLQQSGE<br>LLTRLKQIGALQ-

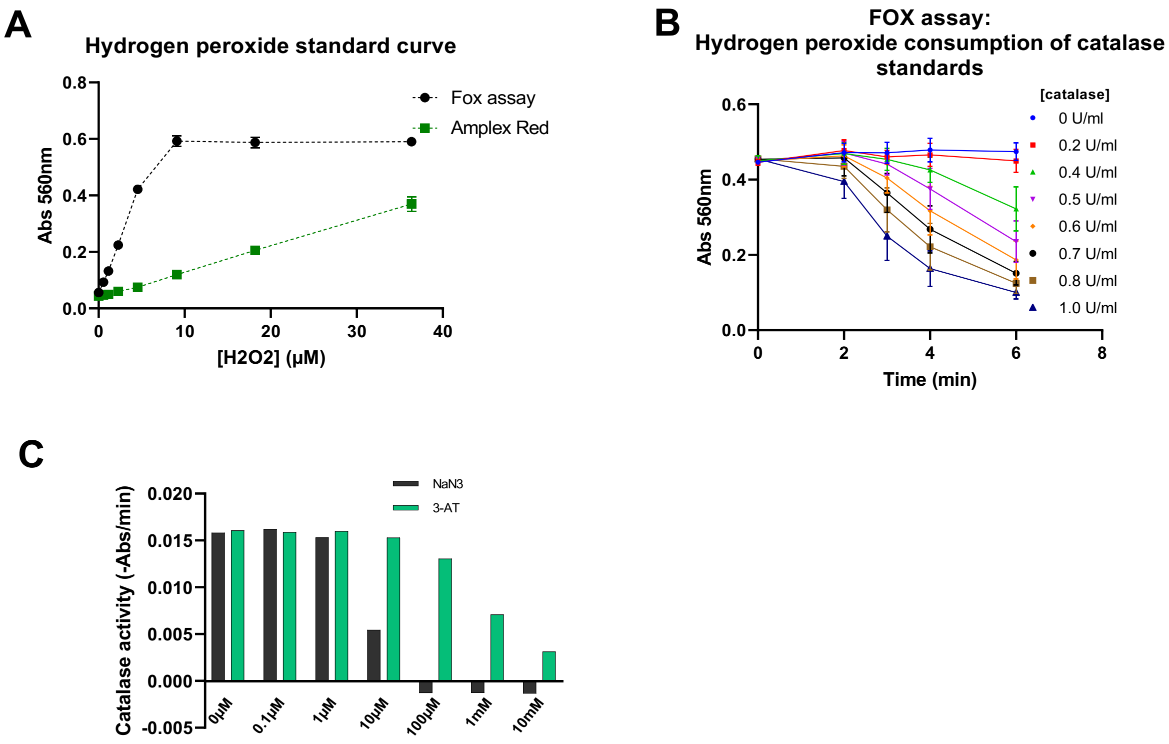

Supplemental Figure S1. A) Hydrogen peroxide standard curves for FOX and Amplex red™ assays. B) Activity assay of different catalase concentrations measured with FOX assay. C) Inhibition of recombinant catalase by 3-AT and sodium azide.

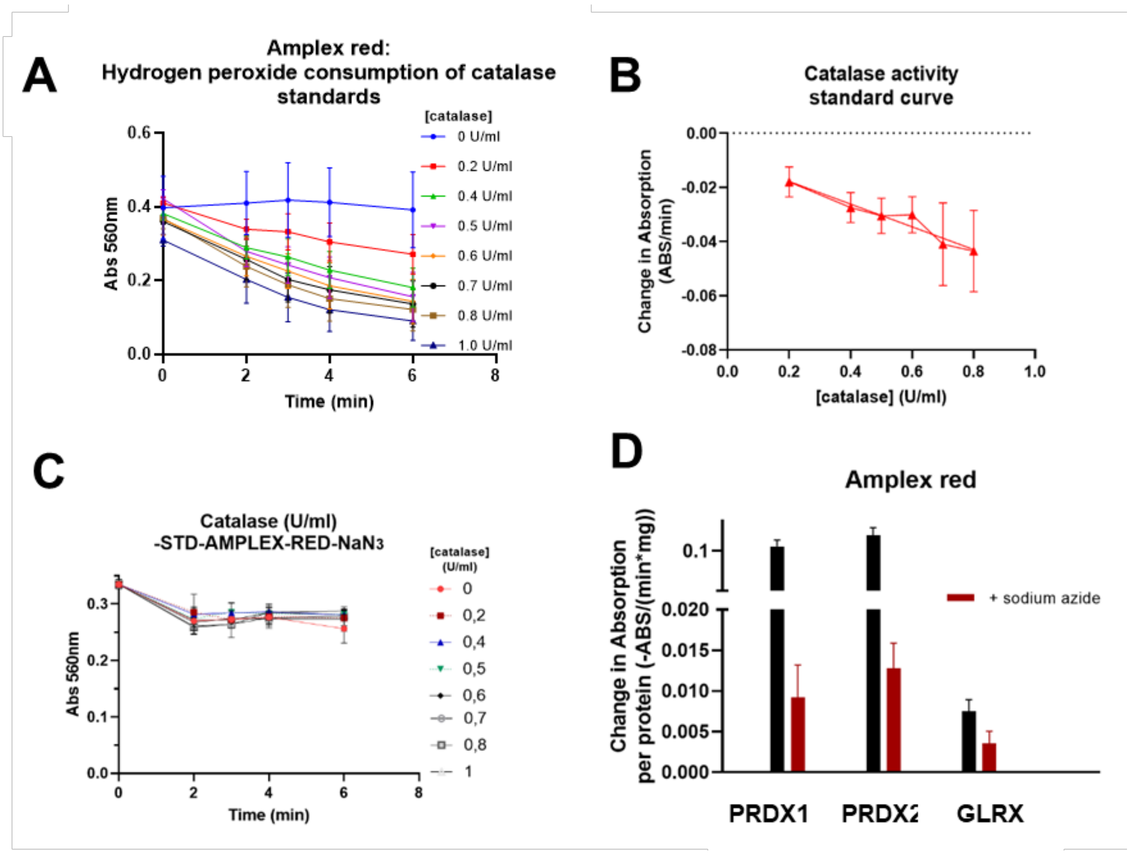

Supplemental Figure S2. A) Activity assay of different catalase concentrations measured with Amplex red™ assay. B) Standard curve for catalase activity (Amplex red™ assay) measured from the linear part of kinetic curves in A. C) Inhibition of standard catalase concentrations by sodium azide. D) Quantification of catalase contamination in recombinant proteins measured using Amplex red™ assay.

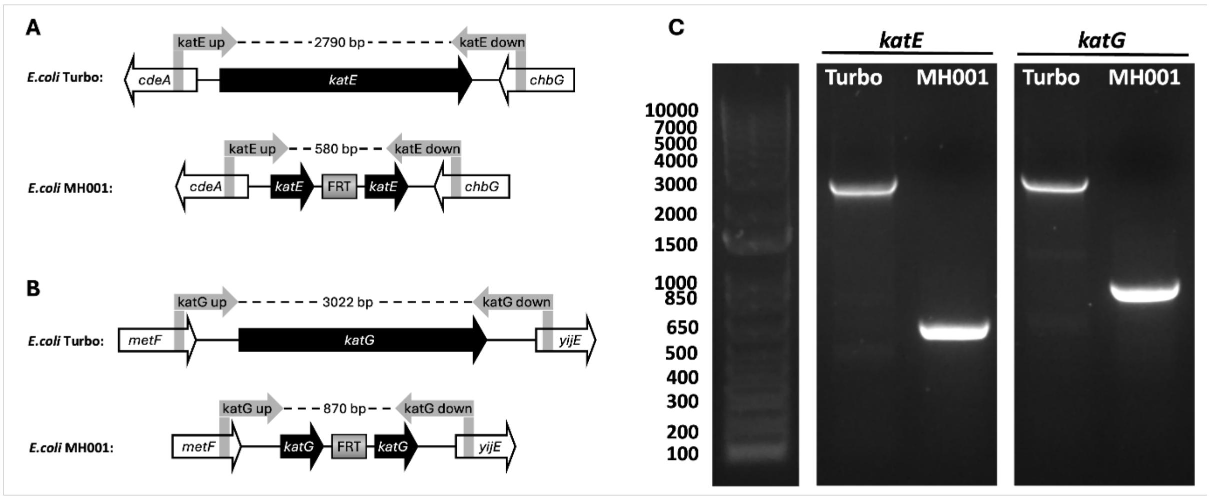

Supplemental Figure S3. Genotyping of the *katE* and *katG* loci by diagnostic colony PCR. Schematic of the genomic organization of the *katE* (**A**) and *katG* (**B**) regions in the wild-type *E. coli* Turbo strain (top) and the engineered strain MH001 (bottom). Black arrows denote the catalase ORFs; white arrows indicate flanking genes for *katE* and *katG*. Grey arrowheads mark primer binding sites (“katE up/down” and “katG up/down”). Dashed lines indicate the expected PCR products: Turbo yields ~2,790 bp for *katE* and ~3,022 bp for *katG*; MH001 yields fragments with FRT scar of ~580 bp (*katE*) and ~870 bp (*katG*) respectively. (**C**) Agarose gel of colony PCRs using the primer pairs in (A–B). For each locus, Turbo produces the larger wild-type band (~3 kb), whereas MH001 produces the smaller junction band (~0.6–0.9 kb), confirming the edited configuration at both loci.
